# A novel model of colitis-associated cancer in SAMP1/YitFc mice with Crohn’s disease-like ileitis

**DOI:** 10.1371/journal.pone.0174121

**Published:** 2017-03-16

**Authors:** Paola Menghini, Luca Di Martino, Loris R. Lopetuso, Daniele Corridoni, Joshua C. Webster, Wei Xin, Kristen O. Arseneau, Minh Lam, Theresa T. Pizarro, Fabio Cominelli

**Affiliations:** 1 Department of Medicine, Case Western Reserve University, Cleveland, Ohio, United States of America; 2 Digestive Health Research Institute, Case Western Reserve University, Cleveland, Ohio, United States of America; 3 Department of Pathology, Case Western Reserve University, Cleveland, Ohio, United States of America; Cincinnati Children's Hospital Medical Center, UNITED STATES

## Abstract

Patients with inflammatory bowel disease (IBD) are at increased risk for developing colorectal cancer. Evidence suggests that colonic dysplasia and colitis-associated cancer (CAC) are often linked to repeated cycles of epithelial cell injury and repair in the context of chronic production of inflammatory cytokines. Several mouse models of CAC have been proposed, including chemical induction through exposure to dextran sulfate sodium (DSS) with the genotoxic agents azoxymethane (AOM), 1,2-dymethylhydrazine (DHM) or targeted genetic mutations. However, such models are usually performed on healthy animals that usually lack the underlying genetic predisposition, immunological dysfunction and dysbiosis characteristic of IBD. We have previously shown that inbred SAMP1/YitFc (SAMP) mice develop a progressive Crohn’s disease (CD)-like ileitis in the absence of spontaneous colitis. We hypothesize that SAMP mice may be more susceptible to colonic tumorigenesis due to their predisposition to IBD. To test this hypothesis, we administered AOM/DSS to IBD-prone SAMP and their non-inflamed parental control strain, AKR mice. Our results showed that AOM/DSS treatment enhanced the susceptibility of colitis in SAMP compared to AKR mice, as assessed by endoscopic and histologic inflammatory scores, daily weight loss and disease activity index (DAI), during and after DSS administration. SAMP mice also showed increased colonic tumorigenesis, resulting in the occurrence of intramucosal carcinoma and a higher incidence of high-grade dysplasia and tumor burden. These phenomena occurred even in the absence of AOM and only upon repeated cycles of DSS. Taken together, our data demonstrate a heightened susceptibility to colonic inflammation and tumorigenesis in AOM/DSS-treated SAMP mice with CD-like ileitis. This novel model represents a useful tool to investigate relevant mechanisms of CAC, as well as for pre-clinical testing of potential IBD and colon cancer therapeutics.

## Introduction

Inflammatory bowel disease (IBD), a chronic, relapsing disorder of the gastrointestinal tract, generally consists of two main forms, Crohn’s disease (CD) and ulcerative colitis (UC) [1]. One of the more debilitating consequences of UC is the development of colonic dysplasia and colitis-associated cancer (CAC) [2]. Studies have shown that patients with IBD have a higher risk for colorectal cancer compared to the general population and this risk is directly proportional to the extent, duration and age of onset of the disease [3]. These observations support the association between chronic inflammation and tumorigenesis, but the mechanisms underlying their pathophysiology are still unclear. Increasing evidence suggests that colonic dysplasia and CAC may be the result of intestinal epithelial hyper-proliferation induced to repair the damage to the epithelial monolayer brought on by the presence of chronic inflammation [4]. In addition, infiltration of both innate and adaptive immune cells (e.g., mast cells, neutrophils, macrophages, dendritic cells, natural killer cells, and lymphocytes) facilitates cancer development through the production of mediators that promote carcinogenesis [5–7]. Inflammatory mediators, including cytokines and chemokines, as well as growth factors, accumulate *in situ* during chronic inflammation, enabling the promotion of tumor initiation, angiogenesis, and tumor progression [8, 9].

Several mouse models of CAC have been proposed, including chemically-induced CAC using dextran sulfate sodium (DSS) combined with the genotoxic agent AOM or DHM [10] and genetically manipulated mice. Genetically-derived mouse models have focused on mimicking immune dysregulation (e.g., IL-10 deficient mice), as well as genetic mutation in pathways associated with tumorigenesis (*e*.*g*., APC, p53, Msh2). However, these models rely on genetic mutations that are absent in patients with IBD and challenge normal mice that lack the genetic predisposition, immunological dysfunction and dysbiosis characteristic of the human condition [11].

The aim of the present study was to evaluate the development of colitis-associated tumorigenesis in a well-characterized spontaneous mouse model of experimental IBD using AOM/DSS administration. SAMP1/YitFc (SAMP) mice develop a progressive CD-like ileitis without chemical, genetic, or immunologic manipulation. The resulting ileitis has remarkable phenotypic similarities to human CD, specifically, disease location, histologic features, response to conventional CD therapies, and occurrence of extra-intestinal manifestations [12]. Moreover, SAMP mice do not typically develop spontaneous colitis or any other pathological abnormalities in the colon. Herein, we provide evidence that AOM/DSS-treated ileitis-prone SAMP mice are more susceptible to colitis and colonic tumorigenesis in comparison to their ileitis-free parental AKR/J (AKR) control strain. In addition, SAMP mice treated with repeated cycles of DSS (chronic colitis) without AOM administration show increased severity of colitis as well as the presence of colonic tumors with high-grade dysplasia.

## Material and methods

### Experimental animals

Nine to 12 week-old SAMP, AKR and C57BL6 mice were maintained under specific pathogen-free conditions, fed with standard laboratory chow (Harlan Teklad, Indianapolis, IN) and kept on 12-hr light/dark cycles in the Animal Resource Core Facility of Case Western Reserve University (CWRU). All protocols were approved by the Institutional Animal Care and Use Committee of CWRU.

#### AOM/DSS-induced CAC model

CAC was induced by co-administration of DSS (TdB consultancy, Uppsala, Sweden) and AOM (Sigma-Aldrich, St. Louis, MO) as previously described [13]. The genotoxic agent, AOM, is commonly used for the induction of colorectal cancer in the distal colon of rodents [14]. On the other hand, DSS is one of the most widely used agents for chemical-induction of colitis in experimental models of IBD and leads to an acute colitis with ulcerations [15]. Groups of age- and sex-matched SAMP, AKR and C57BL6 mice (n = 6) were injected intraperitoneally with a single dose of AOM (7.4 mg/kg) dissolved in saline solution. Two weeks later, 3% DSS was added to drinking water for the next 7 days, followed by 2 weeks of regular drinking water. This cycle was repeated once with mice euthanized at day 56. Body weight and DAI were assessed during DSS administration and during the first days of recovery. Endoscopic evaluation was performed at day 0, 33, and 56 in order to assess the level of inflammation, as well as tumor growth and progression. All experiments were repeated at least twice, and for all experiments, microbiota was normalized before DSS-induced colitis as previously described [13].

#### Experimental model of chronic colitis

Chronic colitis was induced as previously described, but with minor modifications [13]. In brief, groups of SAMP and AKR mice (n = 6) were given 3% DSS in drinking water for 7 days followed by 2 weeks of normal water. This cycle was repeated for a total of 3 times (63 days total). During treatment, mice were monitored in order to evaluate body weight and DAI. Colonoscopy was performed prior to euthanasia to evaluate the presence of inflammation and tumor lesions.

### Endoscopy

Colonoscopy was performed using a flexible digital ureteroscope (URF-V, Olympus America, Center Valley, PA) with an 8.5 Fr (2.8 mm) tapered-tip design and a motion range of 180° in an up angle and 275° in a down angle, as previously described [13]. The endoscope system includes a video system center (Olympus America), a xenon light source (Olympus America) and a video recorder (MediCapture, Plymouth Meeting, PA). Video images and pictures were recorded and archived to USB flash memory in an MPEG-2 format and JPG, TIFF, PNG, and DICOM formats, respectively, with a maximum resolution of 1,280 × 1,024 pixels. Colonoscopy was performed on days 1, 33 and 56 of treatment, and inflammation evaluated by a previously validated endoscopic scoring system [16], which incorporates 4 different parameters to assess colonic inflammation: perianal findings (diarrhea, bloody feces or rectal prolapse), wall transparency (ability to observe colonic mucosal blood vessels), intestinal bleeding (spontaneous or procedurally induced by endoscope due to mucosal friability), and focal lesions (edema, erosions and ulcers). Mice were anesthetized via inhalation of 4% isoflurane supplemented with 100% oxygen, USP (Piramal Critical Care, Bethlehem, PA) prior to performing endoscopy, and no colonoscopy preparations, such as fasting or laxatives, were required.

### Histology

Colons were removed from mice, flushed of fecal contents with cold PBS, opened longitudinally, and fixed in Bouin’s solution (Ricca Chemical, Arlington, TX) for 24 hr. Fixed tissues were processed, paraffin embedded, sectioned at 3–4 μm, and stained with hematoxylin and eosin (H&E). All processed sections were subsequently evaluated by a GI pathologist in a blinded fashion.

### Assessment of DAI, total inflammatory scores and colonic tumorigenesis

As previously described, DAI, performed by an experimentalist blinded to the study [17], was evaluated according to the systems of Cooper *et al*. [18] and Hartmann *et al*. [19]. Essentially, to obtain a total clinical DAI ranging from 0 (healthy) to 4 (maximal score for DSS-induced colitis), the average score of (a) body weight loss (*i*.*e*.,—0, none; 1, 1%-5%; 2, 5%-10%; 3, 10%-20%; 4, >20% (b) stool consistency (*i*.*e*.,—0, normal; 2, loose stool; 4, diarrhea, and (c) bloody stool (*i*.*e*.,—0, negative; 2, fecal occult blood test positive; 4, gross bleeding) was calculated for each experimental animal. The presence of blood in stools was tested with the Hemoccult Rapid Diagnostic Test Kit (Beckman Coulter, Pasadena, CA). Similarly, the total inflammatory score was calculated using the sum of 4 individual indices, including the degree of (a) ulceration, (b) re-epithelialization, as well as (c) active and (d) chronic inflammation. Each index was assigned with a number that correlates with the degree of severity in each condition for each experimental animal [17]. The presence of tubular adenoma, low and high grade dysplasia and mucosal carcinoma was assessed according to the WHO classification of tumors of the digestive system [20].

### Isolation and culture of Mesenteric Lymph Node (MLN) cells

MLNs were aseptically removed and gently pressed through a 70-μm cell strainer to obtain single cell suspensions, as done previously [13]. Resulting cells were cultured (1 x 10^6^/ml) in RPMI medium 1640 (HyClone Laboratories, Logan, UT) with 10% FBS and 1x penicillin/streptomycin, in the presence of immobilized anti-CD3 (5μg/ml) (eBioscience, San Diego, CA) and soluble anti-CD28 (1μg/ml) (eBioscence) in a humidified 5% CO_2_ at 37°C incubator for 72 hr. Supernatants were subsequently collected and stored at -80°C for further analysis.

### *In vitro* organ culture

Colon segments were dissected and rinsed with PBS to remove fecal contents and opened longitudinally. Tumor and non-tumor tissue samples were then portioned (30–70 mg/ml/well) into 24-well tissue culture plates (Corning Costar, Lowell, MA) and cultured in complete RPMI 1640 medium (HyClone Laboratories) with 10% FBS and 1x penicillin/streptomycin, for 24 hr. Tissue samples were incubated in a humidified 5% CO_2_ at 37°C. Supernatants were subsequently collected and stored at -80°C for ELISA assays.

### *q*RT-PCR

Mouse colon tissues were placed in RNAlater (Applied Biosystems, Forest City, CA), left at 4°C overnight, then frozen and stored at -80°C. Total RNA was isolated using the RNAeasy kit (Qiagen, Germantown, MD) and converted into cDNA using the High Capacity RNA-to-cDNA kit (Applied Biosystem). mRNA expression of IL-6, IL-10, IL-17A, IL-17Rb IL-22, IL-23, TNF-α, IFN-γ, TGF-β, β-Catenin, COX-2, Cyclin D1, c-Myc, and MMP7 was measured and normalized to mouse 18S rRNA.

### ELISA assay

Ready-Set-Go ELISA kits (eBioscience, San Diego, CA) were used to measure cytokine protein levels in supernatants of *in vitro* anti-CD3/CD28-stimulated MLNs, and were performed according to manufacturer’s instructions.

### Immunohistochemistry

Five μm tissue sections were cut from formalin-fixed, paraffin-embedded blocks. Following de-paraffinization and rehydration, the sections were microwaved twice for 5 min in 10 mM sodium citrate pH 6.0 to unmask cross-linked epitopes. Immediately following antigen retrieval, endogenous peroxidase activity was quenched with 3% H_2_O_2_ in distilled H_2_O. Tissue sections were incubated at 4°C overnight with either a rabbit monoclonal anti-β-catenin antibody (Abcam, Cambridge, MA) at 1:100 dilution, a rabbit monoclonal anti-MMP7 antibody (Abcam) at 1:100 dilution, or a rabbit monoclonal anti-COX-2 (Abcam) at 1:100 dilution. Sections were then washed and incubated with SignalStain^®^ Boost IHC Detection Reagent HRP, Rabbit (Cell Signaling, Danvers, MA) for 30 min. After washing with PBS, the sections were incubated with 3,3′-diaminobenzidine (Vector Labs, Burlingame, CA), and counterstained with hematoxylin. As a negative control, duplicate sections were immunostained without exposure to the primary antibody. BrdU, 5-bromo-2'-deoxyuridine, staining was also performed as previously described [13]. Three hours prior to euthanasia, mice received a single intraperitoneal injection of BrdU, which is incorporated into replicating DNA and subsequently localized using a specific anti-BrdU monoclonal antibody. After colon tissue fixation, actively replicating cells were detected by immunohistochemistry using the cell proliferation kit by GE Healthcare Life Sciences (Marlborough, MA). BrdU-labeled cells were quantitatively analyzed with the aid of the imaging software MetaMorph (Molecular Devices Corp., Sunnyvale, CA, USA).

### Statistical analysis

All experiments were performed at least twice. Both univariate and multivariate statistical analyses were conducted with the collective data from multiple experiments. Student’s t tests and/or one-way ANOVA were used to compare continuous data across all available experimental groups, provided the data fulfilled the assumptions for parametric statistics. In addition, non-parametric tests were used for data with non-fulfilled distribution assumptions due to some normalized data. Data were expressed as SEMs with ≥ 95% confidence intervals; an alpha level of 0.05 was considered significant. All statistical analyses were performed using GraphPad Prism Version 5 (GraphPad Software, San Diego, CA).

## Results

### SAMP mice with CD-like ileitis develop more severe colitis in response to AOM/DSS treatment

**Fig 1:**
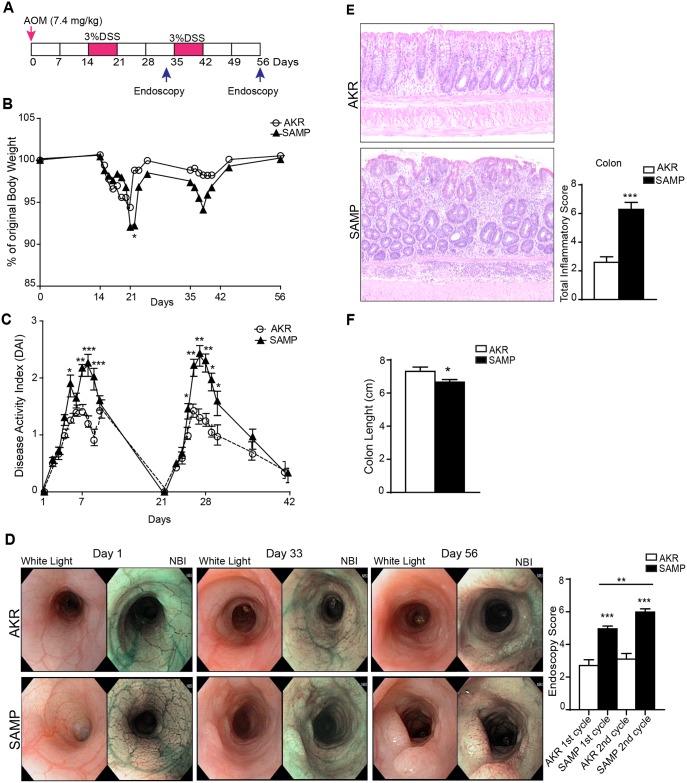
SAMP mice display more severe colonic inflammation after AOM/DSS treatment. Schematic representation of AOM/DSS model (A). Significant increase, both in body weight loss (B) and DAI scores (C), are seen in SAMP compared to AKR mice following multiple DSS cycles. High-resolution endoscopy of distal colons was performed at 3 different time points post-AOM/DSS treatment, *i*.*e*., day 1, 33 and 56, and show signs of colitis and tumor progression following the first and second DSS cycle, particularly in SAMP mice. Increased colitis and tumorigenesis (D) are evident, based on endoscopic scores in SAMP relative to AKR mice. Furthermore, representative photomicrographs of colon sections of both AKR and SAMP mice with histologic scores (E) demonstrate that SAMP mice exhibit more severe colitis. Colon length was measured at the end of treatment (F) and results are presented as mean ± SEM; * *P* < 0.05, ** *P* < 0.01, *** *P* < 0.001; n = 12.

The rodent CAC model was recapitulated in this study using a combination of DSS with the mutagenic agent AOM. The loss of body weight, which is a major clinical indicator of the severity of DSS-induced colitis, was increased in SAMP compared to AKR control mice after each cycle of DSS (Fig 1B). Consistently, the DAI was markedly higher in SAMP compared to AKR mice (Fig 1C). The colon length is considered another macroscopic indicator of the severity of DSS-induced colitis; a shortened colon reflects more severe colitis. As shown in Fig 1F, SAMP mice exhibited a significant shorter colon than AKR mice (AKR = 7.311 ± 0.0.2563 vs. SAMP = 6.667 ± 0.1491; *P* = 0.00490).

To assess the presence and severity of inflammation and tumor progression, endoscopy was also performed. Paired images were acquired using normal light to white balance and narrow band imaging (NBI) vs (normal white light) of diseased colorectal mucosa and other proximal intestinal segments were evaluated. AOM/DSS tumors are better characterized with NBI, particularly during the early stages of development when not protruding into the lumen. At day 0, distal colons in both SAMP and AKR mice are healthy, as depicted by the NBI images, which reveal normal vascularization. After the first cycle of DSS, however, the severity of colitis was increased, and was especially more pronounced in SAMP compared to AKR mice (endoscopic score: AKR = 2.706 ± 0.3607 vs. SAMP = 4.953 ± 0.1768; *P* = 0.0007) (Fig 1D). More interestingly, SAMP mice showed the presence of neoplastic lesions in this early stage of treatment, while AKR mice showed only features of inflammation, such as edema and presence of erosions. Following the second cycle of DSS, the severity of colitis was exacerbated in SAMP compared to AKR mice, with significant tumor progression. The endoscopic score was significantly higher in SAMP compared to AKR controls (AKR = 3.103 ± 0.3391 vs. SAMP = 5.982 ± 0.2022; *P* < 0.0001), and increased continuously during the course of the disease development (endoscopic score: SAMP 1^st^ cycle = 4.953 ± 0.1768 vs. SAMP 2^nd^ cycle = 5.982 ± 0.2022; *P* = 0.0034), whereas no such effect was observed in the AKR group (Fig 1D). Consistent with the increased endoscopic score, the histological analysis of colon sections showed a more severe inflammation in SAMP, which increased in the overall inflammatory scores compared to control mice (AKR = 2.597 ± 0.3841 vs. SAMP = 6.292 ± 0.4852; *P* < 0.0001) (Fig 1E). Untreated SAMP and AKR mice (n = 8) show no evidence of colonic inflammation with histological scores = 0 (data not shown).

### SAMP mice with CD-like ileitis are more susceptible to AOM/DSS-induced colitis-associated tumorigenesis

**Fig 2:**
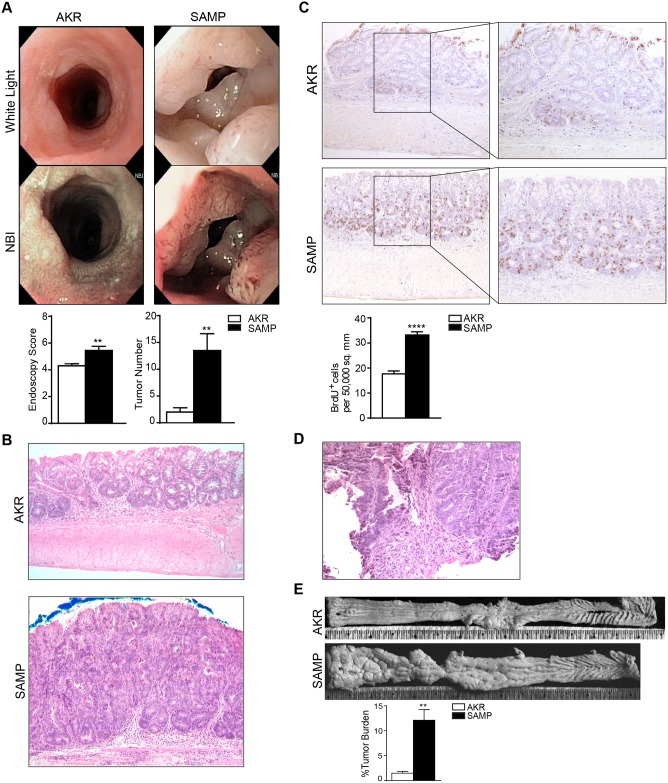
SAMP mice are more susceptible to tumorigenesis after AOM/DSS administration. High resolution endoscopic images of distal colons following AOM/DSS administration revealed remarkable tumorigenesis in SAMP mice (A), as both endoscopic scores and tumor numbers are shown to be increased compared to AKRs. ** *P* < 0.01; n = 12, ** *P* < 0.01; n = 6, respectively. Representative photomicrographs of colon sections stained with H&E showed that SAMP mice develop tubular adenoma with high-grade dysplasia, while AKR only developed low-grade dysplasia (B). Representative photomicrographs of colon biopsy stained with H&E showed that SAMP mice develop intramucosal carcinoma (D). Histological sections treated with BrdU antibody showed that a higher number of cells are actively replicating their DNA (brown staining) in colonic sections from SAMP vs. AKR mice (C). **** *P* = 0.0001. Stereomicroscopic images of colonic tissues showed that SAMP mice had a significantly higher percentage of tumor burden than AKRs (E). ** *P* = 0.0023.

Endoscopic evaluation at the end of the treatment revealed more severe tumorigenesis in the colons of SAMP mice, as demonstrated by higher endoscopy scores and increased numbers of tumors compared to their AKR counterparts (Fig 2A). Microscopically, the colon of AKR mice showed low-grade, non-malignant dysplastic lesions, while SAMP mice showed an increased frequency of tubular adenoma with high-grade dysplasia (Fig 2B). Moreover, histological assessment of biopsy samples collected from colons during endoscopy revealed the presence of intramucosal carcinoma (Fig 2D). The presence of tubular adenoma, low and high grade dysplasia and intramucosal carcinoma was assessed according to the WHO classification of tumors of the Digestive system [20]. BrdU staining displayed a higher level of cell proliferation and DNA replication activity in SAMP colons compared to AKR mice (AKR = 17.68 ± 1.155 vs. SAMP = 33.19 ± 1.284; *P* = 0.0001) (Fig 2C). Finally, stereomicroscopic analysis of colonic tissues showed that SAMP mice had a significantly higher percentage of tumor burden than AKRs (AKR = 1.477 ± 0.3511% vs. SAMP = 12.12 ± 2.131%; *P* = 0.0023) (Fig 2E). Interestingly, compared to C57BL6 mice treated with AOM/DSS, SAMP mice developed a significant increase of tumor number (SAMP = 11.00 ± 2.023 vs C57BL6 = 2.500 ± 0.3416, *P* = 0.0171) (S1 Fig and Fig 1C), even though the total inflammatory score and the endoscopic score didn’t show any significant difference. Altogether, these data indicate that SAMP mice that develop spontaneous CD-like ileitis are more prone to CAC compared to their parental control AKR mice and C57BL6 mice, which are not affected by spontaneous intestinal inflammation.

### SAMP mice displayed an abnormal cytokine profile upon AOM/DSS treatment

**Fig 3:**
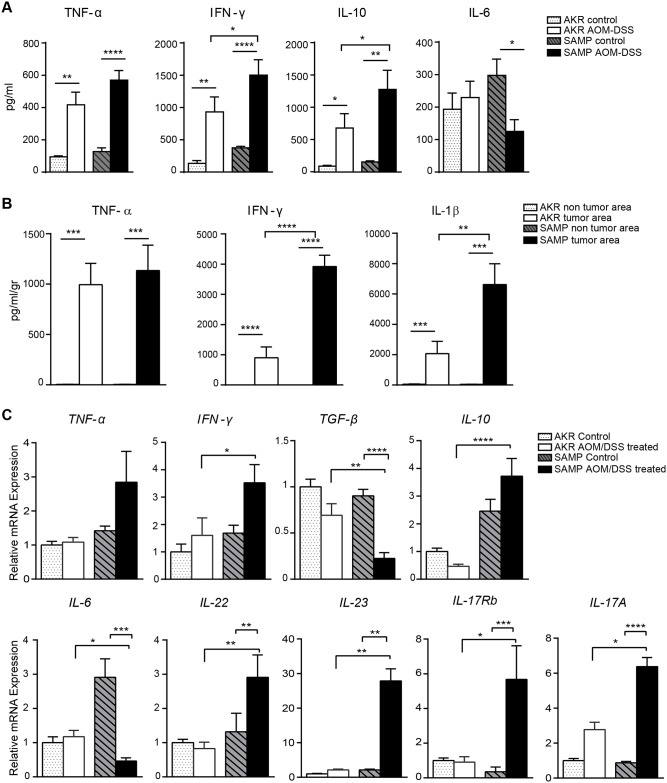
SAMP mice show an abnormal cytokine profile in response to AOM/DSS treatment. MLN cells isolated and incubated for 72 hrs in the presence of anti-CD3 and anti-CD28 antibodies. Cell-free supernatants were analyzed by ELISA. SAMP-derived MLN cells produced increased amounts of IFNγ and IL-10, and lower levels of IL-6 (A) * *P* < 0.05, ** *P* < 0.01, **** *P* < 0.0001; n ≥ 6. IFNγ and IL-1β production from cultured colonic biopsies after treatment with AOM/DSS was higher in SAMP comparing to AKR mice (B) **** *P* < 0.0001, ** *P* <0.01, respectively; n ≥ 8. Cytokine mRNA expression profiles from homogenized colonic tissue samples from untreated and treated SAMP and AKR mice are shown in (C). * *P* < 0.05, ** *P* < 0.01, *** *P* < 0.001; n ≥ 8.

The overproduction of pro-inflammatory cytokines (*i*.*e*., TNFα and IFNγ) plays a key role in cell transformation and malignancy [21]. To further investigate the effects of AOM/DSS treatment in SAMP mice that are prone to chronic intestinal inflammation, cytokine profiles were evaluated. To this end, the production of pro-inflammatory cytokines was measured in cultured supernatants from MLNs and tumor biopsies, as well as from freshly isolated colonic tissues, using ELISA kits and qRT-PCR. MLN cells isolated from SAMP mice and stimulated with anti-CD3 and anti-CD28 secreted elevated levels of TNFα, IFN-γ and IL-10 while IL-6 levels were decreased compared to MLNs from AKR mice (Fig 3A). More importantly, the levels of IFN-γ and IL-10 were higher in AOM/DSS-treated compared to untreated SAMP mice supporting the concept that the induction of CAC in this model further increases cytokine production in MLNs (Fig 3A). SAMP colonic organ-cultured tumor biopsies showed increased levels of IFN-γ and IL-1β compared to AKR mice (Fig 3B). Although the levels of TNFα were significantly increased in the tumors from both strains, no difference was observed when comparing between strains. Furthermore, in areas lacking tumors, basal levels of IFNγ and IL-1β were undetectable. Since the *in situ* accumulation of cytokines is considered one of the major triggers in CAC, we also evaluated cytokine mRNA expression in colonic tissues. AOM/DSS administration increased the colonic mucosal mRNA expression of several key inflammatory cytokines in SAMP compared to AKR control mice, including TNFα, IFNγ, TGFβ, IL-6, IL-10, IL-17A, IL-17Rb, IL-22 and IL-23. In particular, Th17 cytokines showed a profound increase in SAMP compared to AKR mice, suggesting that they may play a key role in tumor formation. IL-10, whose expression is thought to be correlated with tumor promotion, was also significantly increased. TGFβ, which is well known to be involved in tumor suppression pathways [22], was decreased in SAMP mice compared to AKR mice. Interestingly, significantly lower expression of IL-6 in colonic tissue samples from SAMP compared to AKR mice was also observed, supporting the data obtained in MLNs (Fig 3C).

### Expression of tumor markers in SAMP mice is increased

**Fig 4:**
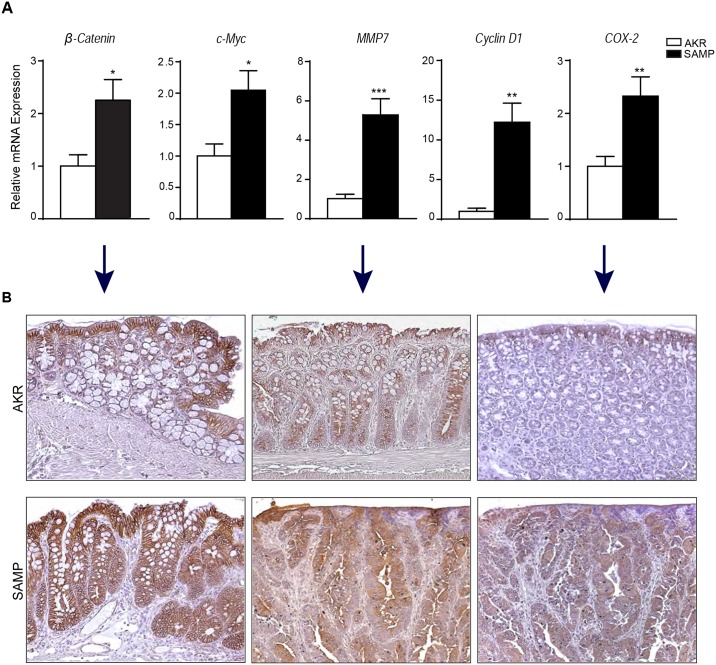
Tumor markers are overexpressed in AOM/DSS-treated SAMP mice. The expression level of several tumor markers, including β-catenin, c-Myc, MMP7, Cyclin D1 and COX-2, were measured by qRT-PCR (A) * *P* < 0.05, ** *P* < 0.01, *** *P* < 0.001; n ≥ 10. Representative photomicrographs of colon sections stained respectively with β-catenin, MMP7 and COX-2 antibodies are shown in (B).

We next evaluated the relative expression of markers implicated in cell cycle events and promotion of colonic tumorigenesis. Fig 4A revealed an abnormal expression of proto-oncogenes, Cyclin D1 and c-Myc, in colonic tissues of SAMP compared to AKR mice (Cyclin D1: AKR = 1.000 ± 0.3919 vs. SAMP = 12.24 ± 2.397; *P* = 0.0015; c-Myc: AKR = 1.000 ± 0.1901 vs. SAMP = 2.046 ± 0.3113; *P* = 0.0103). Alterations in β-catenin expression have also been shown to be important in both CAC and sporadic colorectal cancer [23]. Our findings confirm an increased expression of this protein in SAMP compared to AKR mice (AKR = 1.000 ± 0.2115 vs. SAMP = 2.263 ± 0.3999; *P* = 0.0184). We also found overexpression of MMP7 in SAMP mice (AKR = 1.000 ± 0.2054 vs SAMP = 5.257 ± 0.8432; P = 0.0003); MMP7 has been shown to correlate with tumor invasion and metastasis in several malignancies [24]. Lastly, we evaluated the expression of COX-2, which was significantly increased in SAMP mice (AKR = 1.000 ± 0.1863 vs. SAMP = 2.326 ± 0.3647; *P* = 0.0050). The overexpression of β-catenin, MMP7 and COX-2 are represented in Fig 4B.

### SAMP mice exhibit DSS-induced tumorigenesis in the absence of AOM administration

**Fig 5:**
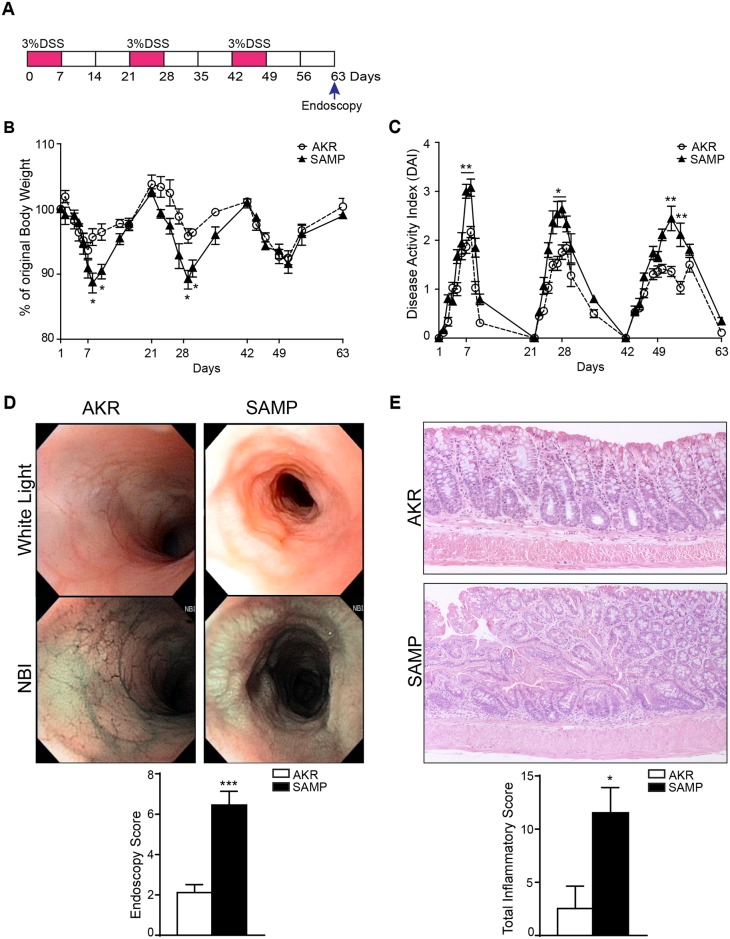
SAMP mice show dysplasia in the absence of AOM treatment after repeated cycles of DSS administration. Schematic representation of DSS-induced colitis in SAMP and AKR mice (A). Body weight loss (B), expressed as the percentage of initial body weight, * *P* < 0.05; n ≥ 6 and DAI scores (C) were higher after chronic DSS-colitis induction in SAMP compared to AKR controls. * *P* < 0.05, ** *P* < 0.01; n ≥ 6. High-resolution endoscopic images with white light and NBI of distal colons together with endoscopic scores (D) (*** *P* = 0.0006; n ≥ 6) and representative photomicrographs of colon sections stained with H&E as well as total inflammatory scores (E) (* *P* = 0.0016; n ≥ 6) are shown for AKR and SAMP mice.

Chronic colitis was induced in the absence of AOM by exposing experimental mice to 3 consecutive cycles of 7 days of treatment with 3% DSS followed by a 14 day recovery period (*i*.*e*., 21 days per DSS/recovery cycle, Fig 5A). Our data showed more severe colonic inflammation in SAMP compared to AKR mice, as evidenced by increased body weight loss and elevated DAI scores (Fig 5B and 5C). Endoscopy revealed features of chronic inflammation in SAMP mice, and surprisingly, the presence of tumors and dysplasia were also observed. By comparison, AKR mice showed minimal visible signs of colonic inflammation after the 14-day recovery period (endoscopy score: AKR = 2.117 ± 0.3953 vs. SAMP = 6.450 ± 0.6854; *P* = 0.0006) (Fig 5D). This finding was confirmed by histological analysis of colons. SAMP mice showed the presence of tubular adenomas and tubular adenomas with high-grade dysplasia, which were absent in the colons of AKR mice (inflammatory score: AKR = 1.000 ± 0.7123 vs. SAMP = 1.628 ± 0.1326; *P* = 0.0016) (Fig 5E). No morphological or histological differences were noticed between tumors developed in SAMP mice after the 3 cycles of DSS comparing to those present in the AOM/DSS model. These results demonstrate that colonic tumorigenesis develops in SAMP mice following repeated intestinal injury induced by exposure to DSS, but in the absence of AOM administration.

## Discussion

An established and strong association between inflammation and cancer has been widely recognized [25]. Epidemiological studies have shown that several chronic inflammatory conditions predispose individuals to different types of cancer; for instance, patients with IBD, in particular UC, have an increased risk for developing CAC [26]. Although several mouse models have been used for the study of IBD and CAC, the majority of these models rely either on genetic alteration of colorectal cancer pathways (*e*.*g*., Wnt–β-catenin or IL-6-STAT3 pathways), modulation of factors involved in the mucosal immune response (*e*.*g*., IL-2 or IL-10 deletion), or treatment with carcinogens, particularly AOM. Such models can be useful to investigate specific aspects of disease pathogenesis, but they do not reflect the complexity of the human condition due to their lack of a predisposing genetic background, as well as presence of immune dysfunction and dysbiosis that are characteristic features of IBD.

SAMP1/YitFc mice represent a well-established model of spontaneous CD-like ileitis. These mice develop intestinal disease without genetic, chemical, or immunologic manipulation, and bear remarkable similarities to the human condition, respond to standard CD therapies, and disease severity worsens over time [12]. SAMP mice develop severe small intestinal disease, but do not display inflammation in the distal colon [27]. The present study was conducted to quantify the effect of AOM/DSS treatment on the induction and progression of colon cancer in SAMP mice in order to test our hypothesis that SAMP mice have more aggressive colitis-associated tumorigenesis due to their natural predisposition to inflammation and IBD. We observed an increased susceptibility to the development of chronic colitis in SAMP mice treated with AOM/DSS compared to their parental control AKR strain. In particular, SAMP mice showed a significant reduction in colon length, as well as increased loss of body weight, DAI and total inflammatory scores. Interestingly, SAMP mice also exhibited more severe tumorigenesis as confirmed by endoscopy. Utilizing stereomicroscopy and histological analysis, we showed that SAMP mice developed a greater number of tumors with increased tumor burden, and that these tumors were characterized by presence of high-grade dysplasia and intramucosal carcinoma, while AKR mice displayed mainly low-grade dysplasia. Altogether, these results indicate that SAMP mice treated with AOM/DSS may represent a more relevant model to investigate pathogenic mechanisms and potential pre-clinical therapeutic modalities to treat CAC.

The development of CAC has been associated with the overproduction of several cytokines, such as TNFα, IFNγ, IL-10, IL-6 and IL-1 [28]. In addition, the IL-23/Th17 pathway has recently been identified to play a critical role in intestinal inflammation and carcinogenesis [29, 30]. Our data are in line with these findings, showing increased production of IL-23/Th17-related cytokines in MLNs and colonic tissues isolated from SAMP mice. In addition, secretion of TNFα, IFNγ, and IL-1β were also found to be increased in organ-cultured tumors isolated from SAMP mice compared to AKR controls. Taking into consideration the increased production of IL-17 and IL-23 [30], which is already known to be associated with a poor prognosis in patients with colorectal cancer, we hypothesize that blockade of the IL-23/IL-17 axis in our SAMP model could decrease the development of intestinal tumors and may have important therapeutic implications. Moreover, TGFβ, which acts as an anti-proliferative factor in normal epithelial cells [31], is decreased in SAMP mice. Interestingly, although other studies have shown a link between the overexpression of IL-6 and the promotion of cancer [32], our data showed that colonic IL-6 production is decreased in SAMP mice. Although the significance of these findings is unknown at the present time, our laboratory is actively investigating the effects of genetic manipulation of TGFβ and IL-6, as well as their pharmacological blockade, in SAMP mice to precisely determine the role of these cytokines in experimental IBD and CAC.

Several proteins, such as β-catenin and MMP7, have been associated in human studies with tumor formation and its progression [33], and it has been suggested that elevated levels of Wnt/β-catenin signaling activity could be critical for the colitis-to-cancer transition [34]. In accordance with these findings, our data showed an increased expression of these markers in the colons of SAMP mice compared to AKR controls. Cyclin D1 and c-Myc are both powerful proto-oncogenes involved in cell cycle regulation. c-Myc has several targets, including genes involved in cell cycle control, apoptosis and DNA metabolism [35]. Cyclin D1 is responsible for the progression from G1 to S phase and has been found to be overexpressed in several types of tumor [36]. In our study, these proto-oncogenes were both significantly increased in SAMP mice. Another factor that appears to favor colorectal tumorigenesis is COX-2 expression, which is associated with both pre-malignant and malignant colorectal tissues [37]. The increase in COX-2 production that we observed in our AOM/DSS-treated SAMP mouse model of CAC is consistent with intrinsic chronic intestinal inflammation and aggressive colonic tumorigenesis observed in these mice. It would be interesting to test the effects of COX-2 inhibitors in our model to determine whether or not this pathway has beneficial or deleterious effects in this model system. Altogether these results further confirm the relevance of colonic tumorigenesis in SAMP mice to CAC in patients with IBD.

Lastly, to isolate the effects of inflammation alone on tumorigenesis in IBD-prone SAMP mice, we induced chronic colitis by administration of repeated cycles of DSS, without pre-treatment with the carcinogenic agent, AOM. SAMP mice displayed a more severe form of chronic colitis compared to AKR controls, as shown by their significant loss of body weight and increased DAI and total inflammatory scores. Surprisingly, even in the absence of AOM, SAMP mice exhibited tubular adenomas with high-grade dysplasia, while AKR mice had no evidence of colonic tumor lesions. This is of particular importance since AOM induces severe DNA damage that is not present in human CAC, making our new model more relevant to the human condition [38].

In conclusion, the present study shows that SAMP mice with CD-like ileitis, but without spontaneous colitis, are more prone to CAC compared to parental control AKR mice and C57/BL6 mice. The use of AOM and DSS in SAMP mice, as herein described, represents a new and reproducible model of severe CAC that takes advantage of a unique mouse strain that has been shown to develop spontaneous chronic small intestinal inflammation without the need for genetic, chemical, or immunologic manipulation. Interestingly, we demonstrate that C57BL6 mice, a strain most often used to study AOM/DSS-induced CAC, also developed tumors; however, the number of tumors is significantly lower compared to SAMP mice (see S1 Fig and Fig 1C). Furthermore, all tumors in C57BL6 mice are histologically graded as low grade tubular adenomas compared to the tumors in SAMP mice, which show a high incidence of high grade dysplasia. Finally, as described in the literature, C57BL6 mice, treated with DSS only up to 3 cycles, do not appear to develop tumors or dysplasia. In contrast, SAMP mice, treated with 3 cycles of DSS, develop tumors in the form of tubular adenomas with high grade dysplasia (see Table 1). Therefore, our new CAC mouse model may be more advantageous as a tool for investigating mechanisms of colonic tumorigenesis and the interplay of intestinal inflammation in its development, further contributing to our understanding of this process and the pre-clinical testing of innovative approaches to human cancer therapies.

**Table 1:** Overall colonic assessment of AKR and SAMP mice following AOM/DSS treatment.

	After 3 cycles DSS		After AOM/DSS	
	AKR	SAMP	AKR	SAMP
Ave. Inflammatory Score (0–15)	0.45 ± 0.28^a^	11.83 ± 1.95^d^	2.70 ± 0.48^b^	6.86 ± 1.07^c^
Endoscopy Score (0–10)	3.00 ± 0.50^a^	6.67 ± 0.33^c^	3.89 ± 0.35^a^	5.60 ± 0.25^b^
Number of Tumors	0.00* ± 0.00^a^	2.00 ± 0.73^c^	1.25¥ ± 0.48^a^	10.42¥ ± 1.93^b^
% of Tubular Adenoma^§^	0	83	50	90
% of HG Dysplasia^§^	0	16	0	70

Average inflammatory and endoscopy scores, number of polyps with percentages of both tubular adenoma and tubular adenomas with high grade (HG) dysplasia are represented for all 4 conditions examined.

*Denotes histologic analysis;

^¥^Denotes stereomicroscopic analysis. Identical superscripts connect variables with nonsignificant difference, whereas different superscripts indicate at least a p value P ≤ 0.05.

^§^The association between the binary outcome and the 4 conditions was examined using chi-squared test for the percentage of the tubular adenoma and HG dysplasia, P = 0.0006; P < 0.0001, respectively.

## Supporting information

S1 Fig SAMP mice have higher number of tumors than C57BL6 mice following AOM/DSS treatment. Total inflammatory scores (A) endoscopic scores (B) and tumor numbers (C) are shown following AOM/DSS together with multiple DSS treatment in both mouse strains. Clearly, the number of tumors is significantly higher in SAMP than in C57BL6 mice. * *P* = 0.0171; n ≥ 10.
(TIF)
