# Supplementary figures and images for: A novel model of colitis-associated cancer in SAMP1/YitFc mice with Crohn’s disease-like ileitis
Source: PLoS One. 2017 Mar 16;12(3):e0174121. doi: 10.1371/journal.pone.0174121 (PMC5354461; doi:10.1371/journal.pone.0174121)

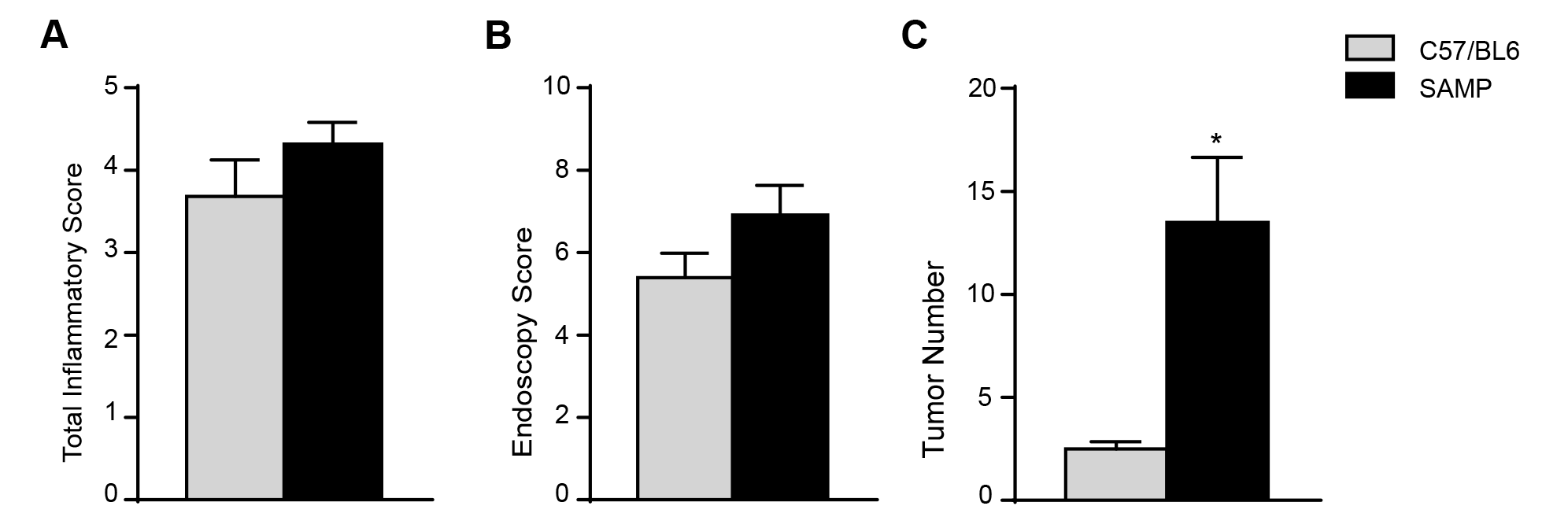

Supplement: S1 Fig — Total inflammatory scores (A) endoscopic scores (B) and tumor numbers (C) are shown following AOM/DSS together with multiple DSS treatment in both mouse strains. Clearly, the number of tumors is significantly higher in SAMP than in C57BL6 mice. * P = 0.0171; n ≥ 10. (TIF) [file pone.0174121.s001.tif]
